# Simultaneous Quantification of Ampicillin and Kanamycin in Water Samples Based on Lateral Flow Aptasensor Strip with an Internal Line

**DOI:** 10.3390/molecules26133806

**Published:** 2021-06-22

**Authors:** Jinbiao Lin, Ang Shi, Ziwu Zheng, Long Huang, Yixin Wang, Honggui Lin, Xuexia Lin

**Affiliations:** 1 Provincial Key Laboratory of Naval Architecture & Ocean Engineering, Marine Engineering College, Jimei University, Xiamen 361021, China; linjinbiao@jmu.edu.cn (J.L.); 13838276619@163.com (A.S.); zwzheng@jmu.edu.cn (Z.Z.); linhongui36@163.com (H.L.); 2 CNTAC Testing Services Co., Ltd., Quanzhou 362700, China; 3 Shanghai WEIPU Chemical Technology Service Co., Ltd., Shanghai 200000, China; cwj516@126.com; 4 Department of Chemical Engineering & Pharmaceutical Engineering, College of Chemical Engineering, Huaqiao University, Xiamen 361021, China

**Keywords:** aptamer, kanamycin, ampicillin, lateral flow assay, water

## Abstract

In this work, a simple and rapid method based on the lateral flow assay (LFA) has been developed for the detection of dual antibiotics. To achieve the quantitative assay and to reduce the non-specific adsorption, an internal system has been developed. A non-specific DNA was exploited as an internal standard and could be recognized by the DNA marker that was coated at the internal line. Two different kinds of aptamers were applied to recognize ampicillin (AMP) and kanamycin (KAM), and the distance between the detection line and conjugate pad was then optimized. Under the optimum conditions, the quantitative assays of AMP (R^2^ = 0.984) and KAM (R^2^ = 0.990) were achieved with dynamic ranges of 0.50 to 500.0 ng/L, and of 0.50 to 1000.0 ng/L, respectively. The LOQs of AMP and KAM were 0.06 ng/L and 0.015 ng/L, respectively. Finally, the proposed method has been successfully applied to analyze aquaculture water, tap water, and lake water, and hospital wastewater, indicating the established method could be used to monitor the environment.

## 1. Introduction

Rapid and sensitive assaying of antibiotic residues is of great significance in food safety, agriculture, and environmental safety, among other areas. Recently, antibiotics in aquatic environments have attracted great attention, as trace levels of antibiotics have been reported to be sufficient to influence human health [1]. Thus, more attention is being paid to the detection of antibiotic residues in the environment, antibiotic transformation, the removal technology, and so on [2,3,4]. According to the reports about antibiotic levels in the aquatic environments in China, the median concentrations of most antibiotics were below 100 ng/L in the surface water and 10 ng/L in the groundwater [1]. For example, AMP had median values that were about 10 ng/L. Thus, great efforts have been made to achieve the sensitive and rapid quantification of antibiotic residues. During the past decade, microbiological inhibition assays [5], capillary electrophoresis [6], high-performance liquid chromatography [7], electrochemistry [8], chemiluminescence [9], lateral flow assay (LFA) and others [10,11] have been widely applied to detect antibiotic residues. Some of these techniques, however, suffer from some drawbacks. The application of the microbiological inhibition assay technique for routine antibiotic residue analysis requires skilled and trained individuals along with sophisticated instrumental setups. In comparison, LFA shows portability and convenience in detection [12,13,14] and enables quantification with high sensitivity. Gao and coworkers developed a third line between the test line and the control line of the LFA to obtain an expansive concentration range for thrombin detection, owing to the reduced hook effect [15]. In order to reduce the ratio of false results, Deyun He and colleagues developed a dual detection mode of an LFA strip that contained surface-enhanced Raman scattering and the colorimetric method for the detection of *Campylobacter jejuni* [16]. Li and coworkers have established a SERS-based LFA for the detection of cypermethrin and esfenvalerate [17]. Similarly, Chen and coworkers developed a near-infrared fluorescence-based LFA for the determination of four kinds of antibiotic residues [18]. Therefore, it is possible to develop a convenient method for the determination of multiplex antibiotic residues with the great advantage of portability.

In recent years, aptamers, a single-stranded DNA/RNA, have been utilized specifically bind to a target with high affinity, and have attracted substantial interest in the application of sensors [19,20]. Aptamers can bind to a broad range of substrates, such as glass [21], nanomaterials [22], and electrodes [23]. Their simplicity and versatility have made aptasensors emerging candidates for LFA strip development. In particular, aptasensors have been readily applied on a cellulose membrane for the fabrication of LFA strips, which holds great potential in on-site application. For the determination of antibiotic residues, a 21-mer nucleotide kanamycin (KAM) aptamer (Kd ≈ 78.8 nM) [24] and three kinds of nucleotide ampicillin (AMP) aptamers (Kd ≈ 10 nM) have been used for the analysis of AMP [25]. In our previous work, a fluorescent-based LFA strip was fabricated for the detection of AMP residue, and a KAM aptasensor was made based on the use of luminescent carbon nanodots [26,27]. In another case, Rozlosnik and colleagues utilized the combination of an aptamer-assisted electrochemical method and microfluidic technology to develop a method for the determination of AMP and KAM [28]. Therefore, it was hypothesized that different aptamers could be applied for quantitative analysis of multiplex antibiotic residues by the application of a third line [15,29].

For the proof-of-concept, AMP and KAM were selected as model analytes because they were the extensively used antibiotics in food products, aquatic products, and clinical drugs. A dual control system (internal strategy and control strategy) was developed for the achievement of excellent quantitative analysis in a fluorescent-based LFA for an antibiotics residue assay. One kind of G-quadruplex fragment was selected as an internal detection DNA because all of these sequences are rich in guanine moieties. The exploitation of the internal line would further reduce matrix interference, hook effect, and cross-reaction, resulting in high accuracy of the assay. In addition, the usage of fluorescence detection resulted from its typical applications, high sensitivity, and the ease of application. The similar intensities of the internal line were achieved in each assay. In the absence of KAM and AMP, the little difference in the initial intensity among the four test lines was set for the decreasing of the discrimination of antibiotics residue. The type of the nitrocellulose (NC) membrane, the distance between each line, the distance between the internal line and the conjugate pad, and the detection time were optimized to get efficient separation. With excellent capability and merits, the quantitative analysis of antibiotics residue was carried out, and the developed method was performed with high sensitivity, selectivity, and repeatability in five kinds of water samples.

## 2. Materials and Methods

### 2.1. Reagents and Materials

Ampicillin trihydrate, bovine serum albumin (BSA), and streptavidin were purchased from Sigma-Aldrich (St. Louis, MO, USA). Electrophoresis gel stain Gold View I type nucleic acid stain, DNA loading buffer, marker I, and DNA ladder were purchased from Beijing Solarbio Science & Technology Co., Ltd. (Beijing, China). Standard products of KAM, AMP, streptomycin sulfate (SMS), oxytetracycline hydrochloride (OTC), deoxytetracycline (DOC), chloramphenicol hydrochloride (CAP), and chlortetracycline hydrochloride (CTC) were purchased from Beijing Solarbio Science & Technology Co., Ltd. (Beijing, China). The NC membrane, a backing card, absorbent pad, and glass fibers were purchased from Jiening Biological Technology Co., Ltd. (Shanghai, China). Absorbent pads were purchased from Whatman-GE Healthcare (Pittsburgh, PA, USA). The Milli-Q water (18 MΩ·cm^−1^) was obtained from a Milli-Q water purification system (Millipore Corporation, Burlington, MA, USA). All chemicals used in this study were analytical grade reagents. All aptamers and oligonucleotides were purchased from Sangon Biotech Co., Ltd. (Shanghai, China). The sequences of aptamers and oligonucleotides are presented in Table 1.

The underlined letters in the internal capture DNA, AMP capture DNA, and KAM capture DNA are partially complementary sequences of the internal HEX-labeled DNA, AMP HEX-labeled DNA, and KMP HEX-labeled DNA, respectively. The italicized bold letters of the AMP and KAM HEX-labeled DNA represent the AMP and KAM aptamer sequences that can be recognized as AMP and KAM, respectively. The bold letters of AMP HEX-labeled DNA and KAM HEX-labeled DNA are the DNA fragments that are complementary with control capture DNAs. Hexachloro-6-carboxyfluorescein (HEX) was used as a fluorescent dye due to its low background signal in the NC membrane.

The stock aptamers and oligonucleotides (100.00 μM), as well as the working solutions of the aptamer, were prepared in 10.00 mM Tris-EDTA (TE) buffer (pH = 8.0). To obtain the special structures, aptamers and oligonucleotides were incubated at 95 °C for 5 min and then slowly cooled down to room temperature. The stock solutions of KAM and AMP prepared by Milli-Q water were 1.00 mg/mL. For quantitative assay, the concentrations of the KAM working solutions were diluted to 0.01, 0.1, 1.0, 10.0, 50.0, 200.0, 500.0, 800.0, and 1000.0 ng/L. The concentrations of the AMP working solutions were diluted to 0.01, 0.5, 5.0, 25.0, 150.0, 250.0, 500.0, and 600.0 ng/L.

### 2.2. Preparation of Streptavidin–Biotinylated DNA Conjugate

The phosphate-buffered saline (PBS, pH = 7.4) and TE buffer were prepared by a standard method. 100 µL of streptavidin solution (2.0 mg/mL) was mixed with 100 µL of 1 µM biotinylated DNA and incubated in the arbitrary shaker maintained at 37 °C for 30 min. Further, the solution was centrifuged in a dialysis tube for 20 min at 6000 rpm under 4 °C, and then dissolved in 500 μL of PBS. The above steps were repeated three times. The remaining solution in a filter was diluted to 500 μL with PBS.

### 2.3. Sample Preparation

Water samples often contain impurities such as particles, proteins, metal ions, and nucleases, among others. To remove and reduce the impurity, the water samples were treated before their analysis. In detail, every sample was centrifuged at 3000 rpm for 5 min, and then the supernatant was collected, then 5 μL of the supernatant was added to 500 μL of trichloroacetic acid and mixed well. After that, every mixture was filtered through a 0.22 μm microporous membrane, and the filtrate was adjusted to pH 7.4 with NaOH solution (1 M) and stored at −20 °C for further experiments. In this work, the addition of trichloroacetic acid to the sample was to make the nuclease and other protein aggregate and precipitate. Further, their interference could be significantly reduced. Based on this sample pretreatment, it could be further used to analyze other samples, which were from different environments or different kinds of samples.

### 2.4. Fabrication of LFA Strip

**Figure 1:**
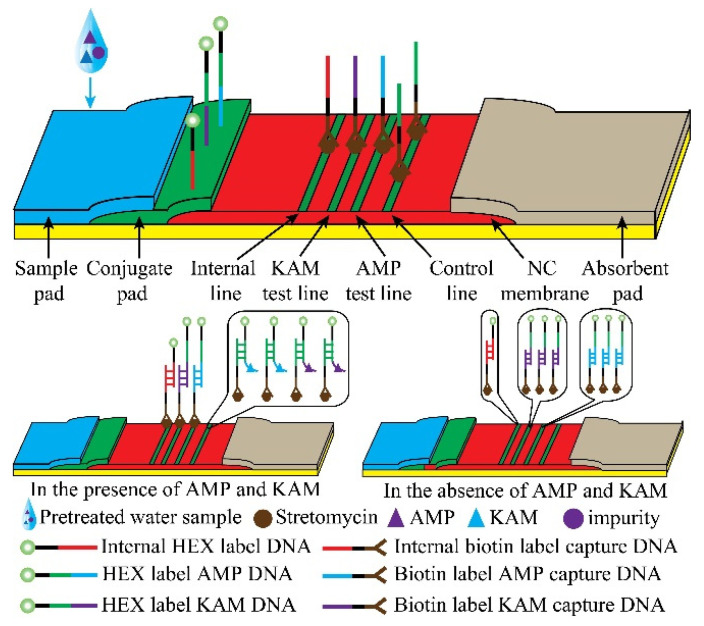
Schematic of lateral flow assay (LFA) for simulation detection of kanamycin (KAM) and ampicillin (AMP).

The structure of the test strip is shown in Figure 1. The conjugate pad, as well as a sample pad, were prepared using glass fiber. The conjugate pad (0.3 × 0.5 cm) was treated with the mixture containing 500 nM AMP HEX-labeled DNA, 500 nM KAM HEX-labeled DNA, 500 nM internal HEX-labeled DNA, and the blocking buffer ((1.5% BSA (*w*/*v*), 0.06% sodium alginate (*w*/*v*), and 1% Triton X-100 (*v*/*v*) to 0.01 M PBS (pH 7.4)), and then was dried at 37 °C overnight. The prepared different streptomycin–biotinylated DNA complex in the coating buffer was separately sprayed on the NC membrane as the internal line, KAM test line, AMP test line, and control line using the SP-20E automatic sampling instrument (Kezhe Biochemical Technology Co., Ltd., Shanghai, China). The coating buffer contained 1% BSA (*v*/*v*) and 10 mM TE buffer (pH 7.4). After drying at 37 °C for 2 h, it was kept at room temperature for approximately 24 h. Further, the NC membrane was stored at 4 °C until use. Finally, the sample pad, conjugate pad, NC membrane, and absorbent pad were pasted on the PVC board to be assembled into the test strip. The arrangement of each part was overlapped 2 mm to ensure the migration of the solution. The assembled pads were arranged in pieces (0.3 × 6.0 cm^2^), installed in the plastic shell, and stored in a desiccator at room temperature.

In a typical antibiotics residue test by LFA assay, 5 μL of saline–sodium citrate buffer containing 0.6 M NaCl, 0.06 M sodium citrate, and 1% BSA (*w*/*v*) as the running buffer was mixed with 10 μL of the pretreated water sample and 5 μL Milli Q water. The mixture was incubated at 25 °C for 1 min before being added onto the sample pad. During the analytical process, the sample solution migrated by capillary force, and the image of the strip was taken after around 10 min by the fluorescence and chemiluminescence imaging system.

### 2.5. Data Analysis

The samples were analyzed by the developed LFA strip, and the intensities were measured. Calibration curves were obtained by plotting *Y* against the concentration of an analyte, in which the value of *Y* was calculated according to the formula:

(1) $$Y=\frac{I}{I_{0}}$$

where *I*_0_ is the intensity at the internal line, and *I* is the intensity at KAM or AMP test line. The limit of detection (LOD) of the analyte was calculated by the background (blank) value + 3 × standard deviation (SD) of this value, and the limit of quantitation (LOQ as the average value of the zero concentration of target (blank) + 10 × SD.

## 3. Results and Discussion

### 3.1. Principle of Aptasensor-Based LFA for Simultaneous Detection of AMP and KAM Residues

The qualitative analysis of antibiotic residues in a given sample is commonly performed by employing chromatography techniques. However, the on-site analysis of antibiotic residues is particularly required for food monitoring and environmental monitoring, such as in hospital sewage waste, livestock and aquaculture wastewater, and industrial wastewater monitoring. A dual control system (internal signal and control signal) was developed for quantitative analysis of antibiotic residues in this work. The internal system was exploited based on one kind of G-quadruplex fragment. The similarity of the DNA sequence can effectively inhibit the matrix effect and the hook effect. The length of the G-quadruplex fragment was much shorter than the AMP HEX-labeled DNA and KAM HEX-labeled DNA. Therefore, the internal HEX-labeled DNA could be easily separated, and cross-reaction could be avoided. In addition, the aptamer could easily recognize and identify the targets with only one functional group, such as the methyl group [30,31], which can further improve the accuracy of the analysis. The similar intensities of test line are also suggested to reduce the signal discrimination.

The internal line LFA strip for the simultaneous detection of AMP and KAM, and the test system consisted of one internal line, two test lines, and one control line, which are shown in Figure 1. The four lines were coated with streptavidin-biotinylated DNA oligonucleotides by using a streptavidin−biotin reaction. The internal line was the first line, the KAM test line was the second test line, the AMP test line was the third test line, and the control line was the fourth line. All captured DNAs were pre-immobilized on the NC membrane. Simultaneously, the conjugate pad was immersed in a molar ratio of 1:1:1 mixture solution that included the same concentration of the internal HEX-labeled DNA, AMP HEX-labeled DNA, and KAM HEX-labeled DNA. In the presence of AMP and KAM, the internal HEX-labeled DNA hybridized with its corresponding target to form the complexes. The complexes flowed along the strip, and then they were captured on their corresponding test line. The free KAM and AMP HEX-labeled DNA were captured on the second and third test line, respectively. The AMP−DNA complex and KAM−DNA complex were captured on the control line. In the absence of AMP and KAM, the internal HEX-labeled DNAs were captured on the internal line; the KAM and AMP HEX-labeled DNA were also captured on the second and third test line, respectively. The remaining KAM and AMP HEX-labeled DNA were captured on the control line. The intensities on the control line were inverse to those of the KAM and AMP lines. Since the signal of the internal line was very little affected by the level of the targets, the internal line was used as an internal signal for quantitative analysis. With the combination of DNA immune competition (control system) and the internal system, the LFA was successfully implemented for highly accurate qualitative analysis of KAM and AMP. Although the use of the internal line-assisted LFA in analyzing dual antibiotic residues is demonstrated here, this approach could be applied in more than two kinds of antibiotic residues and other molecules.

### 3.2. The Internal Line for Dual Antibiotics Residue Analysis

**Figure 2:**
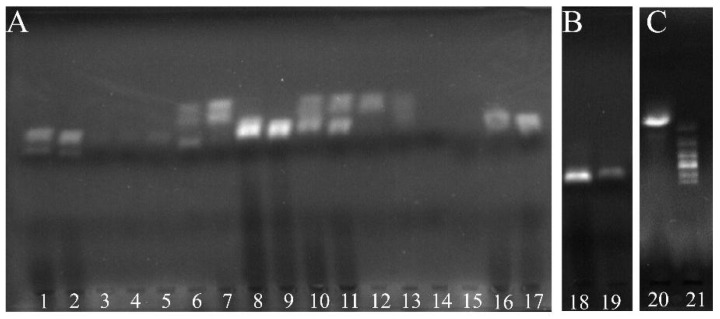
The internal line for KAM and AMP assay by 2% agarose gel electrophoresis. (**A**) The interactions among oligonucleotides. (**B**) The interaction between the internal capture DNA and the internal detection DNA. (**C**) Internal Hex-labeled DNA and ladder. 1~21 mean Internal capture DNA–AMP Hex-labeled DNA, Internal capture DNA-KAM Hex-labeled DNA, Internal capture DNA–AMP, Internal capture DNA–KAM, Internal capture DNA, Internal capture DNA–Internal Hex-labeled DNA, Internal Hex-labeled DNA, AMP Hex-labeled DNA, KAM Hex-labeled DNA, Internal Hex-labeled DNA–AMP Hex-labeled DNA, Internal Hex-labeled DNA–KAM Hex-labeled DNA, Internal Hex-labeled DNA–AMP, Internal Hex-labeled DNA–KAM, AMP, KAM, AMP Hex-labeled DNA–AMP complex, KAM Hex-labeled DNA–KAM complex, Internal capture DNA–Internal Hex-labeled DNA, Internal capture DNA, Internal Hex-labeled DNA, DNA ladder.

The purpose of this work is to quantitatively analyze KAM and AMP at the same time. The most critical element is the added internal system, and the internal DNA should not affect the target analysis. Hence, the internal line was investigated by 2% agarose gel (Figure 2A). It shows that the complex of the internal capture DNA and internal HEX-labeled DNA was migrated at the fastest rate, while the AMP Hex-labeled DNA−AMP complex was second, and KAM Hex-labeled DNA−KAM complex was third. The experimental results show the internal system was slightly affected by other DNAs, demonstrating that the internal system was well developed. For the achievement of high sensitivity in detecting KAM and AMP, the internal was set as the first test line, while the KAM and AMP test lines were set as subsequent lines, respectively. To accumulate more levels of the AMP Hex-labeled DNA−AMP complex on the test line, the control test line was set as the fourth line. Although the introduction of AMP and KAM could accelerate the migration speed, promote diffusion, and change the electronegativity of DNA, the gel graphs also show there were few impurity bands, indicating slight cross-reactions happened. These results implied that the designed internal capture DNA and the internal Hex-labeled DNA, and other kinds of DNAs, are suitable for the qualitative and quantitative analysis of AMP and KAM residues.

### 3.3. Optimization of LFA Assay Parameters

**Figure 3:**
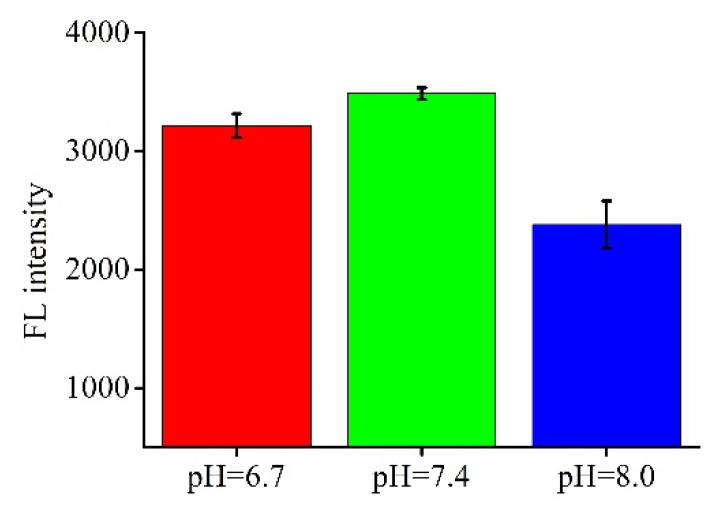
The effect of PBS buffer pH on the fluorescence intensity.

The pH value of buffers is important in this assay because it relates to the efficiency of the assay. Different kinds of PBS buffer (pH = 6.7, 7.4, pH = 8.0) containing 20 mM KCl and 300 mM NaCl were studied (shown in Figure 3). The results show that PBS buffer (pH = 7.4) was suitable for this system because the strongest fluorescence intensity was achieved. Thus, the next experiment was taken with PBS buffer (pH = 7.4). Furthermore, the concentration of detection probes was another key factor in the accuracy of the assay. In our previous work [27], it was found that 500 nM was the most suitable, so then 500 nM of detection probe was chosen in this work.

**Figure 4:**
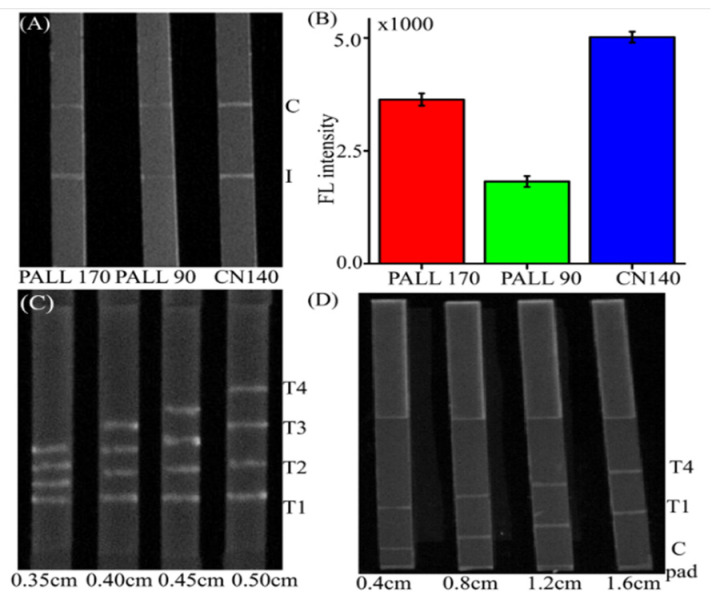
Optimization of LFA assay parameters. (**A**) The image of the NC membrane type. (**B**) The corresponding FL intensity of the different NC membrane type. (**C**) The distance between the test lines. (**D**) The distance between the internal line and the conjugate pad. Experiments were carried out, three experiments in parallel. T means test line. C pad presents the conjugate pad. Each experiment was carried out three times in parallel.

To achieve the optimal conditions for the quantitative determination of AMP and KAM, the type of NC membrane, the distances between each test line, the distances between the internal line and the conjugate pad, and the detection time were investigated. The capture DNAs were immobilized on their corresponding lines. In the rapid analysis of immune-chromatography, the property of the NC membrane has a significant impact on the chromatographic separation, the sensitivity, false-positive signals, and so on. If the migration speed is rapid, the reaction will be inadequate, resulting in the decreasing of the accuracy of the assay. When the chromatography speed is too slow, the result is the increase of analysis time. Hence, the type of NC membrane was optimized. As shown in Figure 4A,B, the signals in the CN140 membrane were clear, and the analytical time was about 2 min. The signals in PALL170 were not as clear as with CN140, and the analytical time was more than 4 min. Furthermore, the PALL90 has shown a short brightness-maintained time. Therefore, CN140 was chosen as the NC membrane for subsequent experiments.

The distance between each line was studied. If the distance between each test line was too close, it might cause insufficient separation and interfere with the capture of other DNAs. Nevertheless, if the distance was too far, the NC membrane and absorbent pad would be extended, and the detection time would increase. Therefore, it was essential to study the distance between each line. Firstly, the distance between each test line was set to be 0.35, 0.40, 0.45, and 0.50 cm. Figure 4C shows that all of these distances present good intensity stability and separation efficiency. However, by considering the limited length of the NC membrane and the feasibility of operation, 0.40 cm was chosen as optimum. Then, the distance between the conjugate pad and the internal line was studied. It was set around 0.40, 0.80, 1.20, and 1.60 cm, respectively. Figure 4D shows the excellent separation was obtained when the detection time was at 5 min. The evidence shows that the higher intensities were obtained when the distance was 0.8 cm or 1.2 cm. Therefore, 0.8 cm was chosen as the distance between the internal line and conjugate pad for balancing the intensity and the length of the NC membrane.

**Figure 5:**
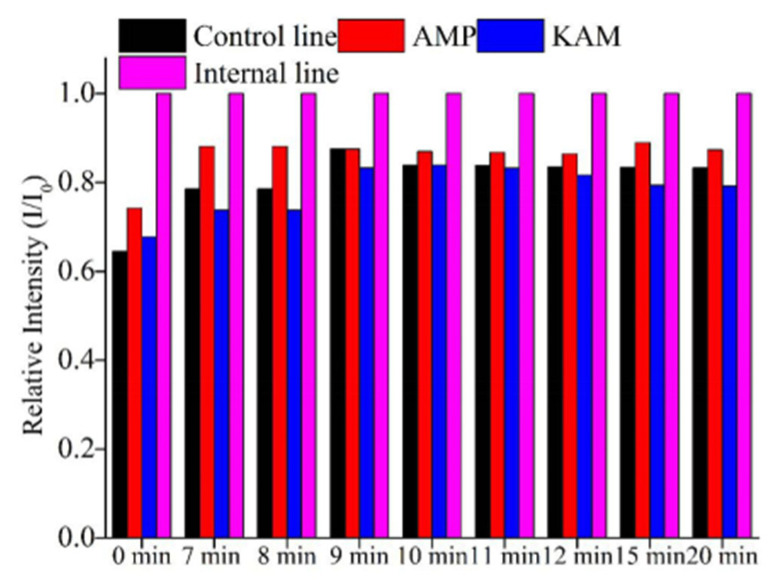
Optimization of the detection time by LFA strip.

After sample injection, the capture DNAs encountered HEX-labeled DNA, and then the complex accumulated in the corresponding test lines, which displayed the increasing FL signals. However, owing to the porosity of the NC membrane, the liquid on it would evaporate gradually with the increasing of time, and the HEX also had time lines. To obtain accurate and highly sensitive results, it is often necessary to determine the efficacy at a specified time. The results found that the intensities increased from 0 to 7 min, and the brightness could be maintained more than 10 min (Figure 5). The intensities of AMP were changed slightly from 15 min to 20 min. However, the detection time was selected at the point of 10 min for saving time.

### 3.4. Specificity

**Figure 6:**
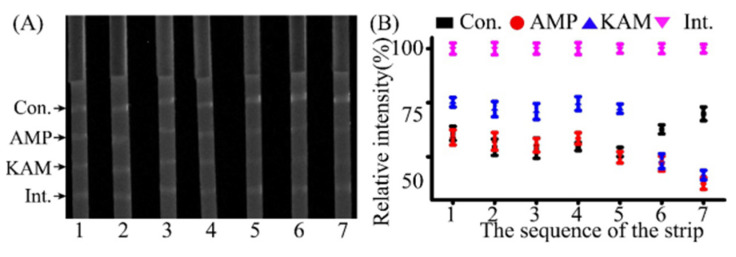
Specificity analysis of the aptamer-based LFA assay for KAM and AMP detection. (**A**) LFA fluorescent images for specificity analysis and (**B**) its corresponding intensities. The concentrations of KAM and AMP on strip 6 were 30 ng/L, and the concentrations of KAM and AMP on strip 7 were 80 ng/L, respectively. The concentrations of SMS, OTC, DOC, CAP, and CTC on strips 1, 2, 3, 4, and 5 were 80 ng/L, respectively. Con. and Int. represent the control line and internal line, respectively.

To demonstrate the specificity of the assay, different kinds of antibiotics were added to samples, and then the selectivity of the developed method was analyzed. Two kinds of AMP and KAM concentrations were chosen to be 30 and 80 ng/L, and the others were set at 80 ng/L. Figure 6A shows that the intensity was almost unchanged in the presence of the other antibiotics. Nonetheless, the intensities were significantly varied with the change of target concentration. Together, our findings indicated that the developed system had shown high specificity for the determination of AMP and KAM.

**Figure 7:**
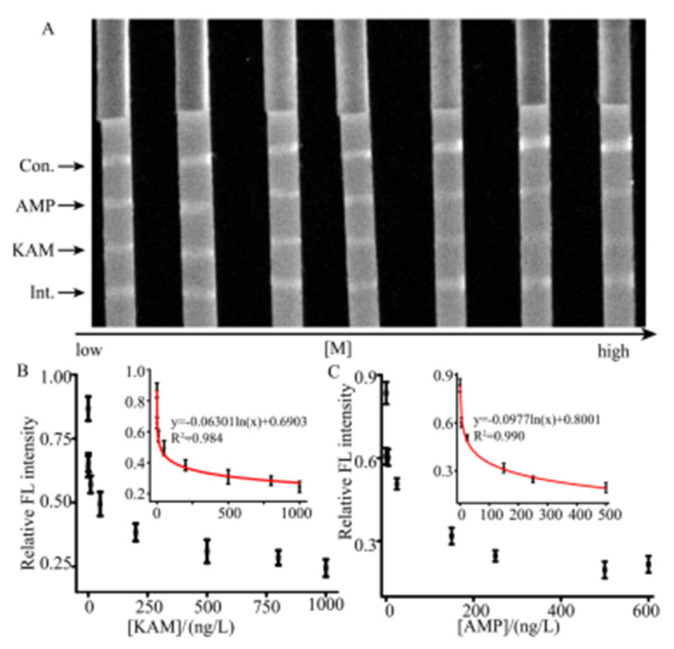
The different AMP and KAM concentrations were used to demonstrate the capability of the developed LFA strip for the quantifications of AMP and KAM. (**A**) Image of the intensity corresponding to AMP and KAM concentrations. (**B**) The quantitative analysis curve for the different KAM concentrations. Inset shows a range from 0.5 to 500 ng/L. (**C**) The quantitative analysis curve for the different AMP concentrations. Inset shows a range from 0.1 to 1000 ng/L.

Based on the utilization of the internal line and immune competition method, a series of the AMP and KAM concentrations were used to demonstrate the capability of the developed LFA strip. Figure 7A displays the image of the strips with different concentrations of KAM and AMP. Figure 7B,C shows the equation is y = −0.06301ln(x) + 0.6903 (R^2^ = 0.984) and the LOQ is 0.015 ng/L for KAM. For AMP, the regression equation is y = −0.0977ln(x) + 0.8001(R^2^ = 0.990), where y represents the relative intensity that is calculated with the ratio of test line to the internal line. The LOQ for AMP is 0.06 ng/L. X represents the concentration of AMP or KAM. The reproducibility was obtained by triplicate measurement at different target concentrations with the relative standard deviations (RSDs) in the range from 2.89% to 6.70%. Our assay system demonstrates sensitivity that is comparable to that of the other developed assays [26,32]. The results implied that the developed method was well suited for the determination of trace levels of antibiotic residues. To further evaluate the quantitative detection of the developed LFA strip, the developed method was compared with the LC-MS, a standard test method for the analysis of antibiotics residue. The results indicated that the developed method possessed more sensitivity than LC-MS (LOQ values of AMP and KAM were around 1.0 × 10^3^ ng/L).

### 3.5. Real Sample Analysis

To demonstrate the feasibility of the developed method, hospital wastewater, chicken farm wastewater, tap water, and aquaculture water were collected and analyzed. The results of the real samples are shown in Table 2. In the hospital wastewater, the intensities of AMP and KAM were detected, but the concentration was below the corresponding LOQs, indicating that AMP and KAM may be commonly used in clinical diagnosis. Similarly, AMP residue was found in the chicken farm wastewater, but below the corresponding LOQs. The results showed that the recoveries of AMP and KAM in hospital wastewater (≤95.65%) and chicken farm wastewater (≤96.51%) were lower than those in other water (≥99.53%). It could be deduced that the transport of antibiotics in aquaculture water and tap water is an effective method for removing the antibiotic contaminants because antibiotics would be adsorbed, hydrolyzed, photolyzed, and biodegraded, among others, during their transport [33,34]. Furthermore, the high recovery and low RSD % indicated that the established method possesses a great selectivity and anti-interference capability, which could detect AMP and KAM in environmental water.

## 4. Conclusions

In summary, a LFA strip was developed with an internal system and control system to determine the trace levels of duplex antibiotics (KAM and AMP). The duplex capability and high sensitivity were achieved by introducing aptamers with fluorescence detection technology. The application of the internal line could reduce the hook effect and the matrix effect. Widely quantitative ranges were achieved for KAM, from 0.5 to 500 ng/L, and for AMP from 0.1 to 1000 ng/L. Based on the internal line, LOQs of KAM and AMP were 0.015 and 0.06 ng/L, respectively. Moreover, it should be noted that the proposal is not limited to KAM and AMP analysis but could be extended to the analysis of other small molecules. Although the capacity of multiplex compounds detection still needs to be improved, the developed method for duplex antibiotics is potentially useful for environmental monitoring, food safety, and medical diagnostics.

## Figures and Tables

**Table 1:** The sequence of aptamers and oligonucleotides used in this work.

Name	Sequence
Control capture DNA	5′-GTCAGATGAATTCGTGTGAGAAAAA-bio-3′
AMP capture DNA	5′-CCGCTATACAACCGCCCG-C6-bio-3′
KAM capture DNA	TGGGGGTTGAGGCTAAGCCGATT TTT-C6-bio-3′
Internal capture DNA	5′-ATTCGTGTGAGAAAA CCAACCCGCCCTACCCA AAAGTCAGATGA-bio-3′
AMP HEX-labeled DNA	5′-*GCGGGCGGTTGTATAGCGG*TTTTTTCTCACACG**AAT TCATCTGAC**-HEX-3′
KAM HEX-labeled DNA	5′-*TCGGCTTAGCCTCAACCCCA*TTTTTT**AATTCA TCTGAC**-HEX-3′
Internal HEX-labeled DNA	5′-GGGTAGGGCGGGTTGGG-C6-Hex-3′

**Table 2:** Concentrations of AMP and KAM residues in water samples (*n* = 3).

Sample	AMP (ng/L)	KAM (ng/L)	Spiked [AMP] (ng/L)	Recovery of Spiked AMP (%)	RSD (%)	Spiked [AMP] (ng/L)	Recovery of Spiked AMP (%)	RSD (%)	Spiked [KAM] (ng/L)	Recovery of Spiked KAM (%)	RSD (%)	Spiked [KAM] (ng/L)	Recovery of Spiked KAM (%)	RSD (%)
Hospital wastewater 1	<LOQ	<LOQ	0.5	93.21	2.32	200	95.51	1.32	1.0	95.81	3.28	200	95.18	1.21
Hospital wastewater 2	<LOQ	ND	0.5	93.65	3.89	200	95.65	2.12	1.0	95.65	3.59	200	95.21	2.42
Chicken farm wastewater	<LOQ	ND	0.5	94.34	2.58	200	96.37	2.25	1.0	95.33	3.36	200	96.51	2.35
Tap water 1	ND	ND	0.5	96.53	3.35	200	99.53	3.27	1.0	98.63	2.21	200	99.78	1.10
Aquaculture water	ND	ND	0.5	97.66	3.08	200	99.66	1.01	1.0	98.95	2.34	200	99.59	1.33

<LOQ, below limit of quantification, ND, not detected.

## Data Availability

The data presented in this study are available on request from the corresponding author.

## Sample Availability

Samples of the compounds are not available from the authors.
